# Fractures of the thoracolumbar spine in osteoporosis

**DOI:** 10.1007/s00068-024-02625-5

**Published:** 2024-09-10

**Authors:** Radko Komadina, Frank W. Bloemers, Marko Jug, Klaus W. Wendt, Christoph Nau, Hans-Christoph Pape

**Affiliations:** 1 University of Ljubljana, Ljubljana, Slovenia; 2 Splošna Bolnišnica Celje, Celje, Slovenia; 3 Amsterdam University Medical Centers, Amsterdam, Netherlands; 4 Ljubljana University Medical Centre, Ljubljana, Slovenia; 5 University Medical Center Groningen, Groningen, Netherlands; 6 University Hospital Frankfurt, Frankfurt am Main, Germany; 7 University Hospital of Zurich, Zurich, Switzerland

**Keywords:** Thoracolumbar spine, Injury, Osteoporosis, Treatment

## Abstract

Due to increasing life expectancy, the prevalence of fractures caused by osteoporosis is raising. These fractures significantly reduce the quality of life in the elderly population. They represent both a disease and an injury simultaneously. While they were once treated solely with conservative methods, new techniques and implants are expanding the indications for surgical treatment. This article presents the current treatment options.

## Introduction

Fractures due to osteoporosis are at epidemic proportions worldwide. The number of elderly people with osteoporosis will increase by 32% in the USA between 2010 and 2030 [1]. The prevalence of spinal vertebral fractures in adults over 40 years of age is 5.4%, rising to 18% in people over 80 years of age. A vertebral compression fracture (VCF) can trigger a vicious cycle of pain and immobility, lead to worsening comorbidities, impair respiratory function, and increase the risk of death by 72% over a five-year period and by as much as 90% over a seven-year follow-up period in the very elderly [2].

Osteoporotic thoracolumbar spinal fractures are a significant source of morbidity in the elderly population, accounting for up to 20% of all osteoporotic fractures. These fractures can lead to significant pain, deformity, and impaired mobility, which can have a major impact on quality of life. Conservative treatment has traditionally been the first line of management for these fractures, but recent advances in minimally invasive surgical techniques have led to a growing interest in their use as a potential alternative.

## Osteoporosis

Bone rigidity helps it resist external deformation and is made up of an inorganic mineral component, which is brittle but resists compressive forces, and an organic component, collagen, which gives the bone its elasticity. Bone toughness is described as the sum of the mineral component of the bone (bone brittleness) and the collagen (elasticity) and resists an external force leaning on it and deforming it. Bone strength is a joint function of bone mineral density, bone turnover, remodelling activity and the microarchitectural arrangement of the bone matrix, but it is also a property of the “material” (mineralisation rate), the denaturation of collagen, and the ability to repair microfractures in the matrix (trabecular microcracks). Bone strength therefore combines bone quantity, measurable by densitometry (DXA), and bone quality, measurable histomorphometrically, by micro-QCT, by some ultrasound experimental methods, and so on.

Nevitt’s coefficient of bone stiffness has the load on the bone from an external force (a fall from standing height, gravity) in the numerator and bone stiffness in the denominator. Fractures due to osteoporosis are therefore understood as both an accident and a disease since the fraction of the Nevitt coefficient of bone strength explains the fracture as an accident (a fall from standing height) in the numerator and as a disease (deformation of the bone due to reduced bone strength) in the denominator [3].

The components of the spinal vertebrae are mainly subjected to compressive forces and, to a lesser extent, to tensional and torsional (bending and stretching) forces. Approximately half of the load is due to the forces exerted by the muscles and ligaments that hold the body upright, and the other half is due to the body's weight. Additional loads are caused by the ongoing activities of each day.

**Fig. 1:**
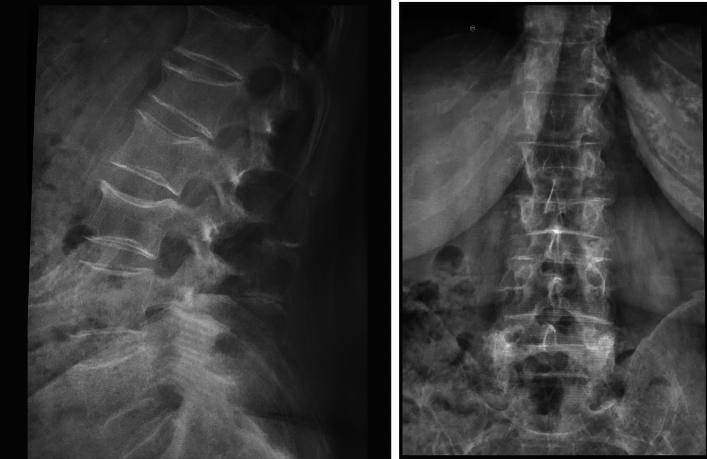
Osteoporotic fractures without known accident

Even in spongiosis of the spinal vertebrae, porosity starts to appear in a graded pattern, with deformities occurring continuously or after a sudden jerking effort. As the porosity gradually increases, the spinal vertebra behaves like a ball of wet snow: the more you squeeze it in your hands, the smaller it gets, but at the same time, the firmer and more compact it becomes. Gravity continuously densifies the vertebra to the density needed to support the weight of the body above it. The middle thoracic vertebrae (widow's hump) and the vertebrae of the lumbar spine are the most fractured. This is a bimodal frequency distribution of thoracic and lumbar spine fractures in osteoporosis in the third stage of life and is due to the biomechanical properties of these two regions of the spinal column. In the plane of the eighth thoracic vertebra (T8), the thoracic kyphosis is most pronounced; therefore, the additional flexion loads are the greatest. The dynamic component of the weight-bearing force predominates. Osteoporotic vertebral fractures can take three forms: compression fractures, in which the height of the whole vertebra is reduced (static component of the weight-bearing force); wedge fractures, which are most common in the mid-thoracic spine and are when the vertebra collapses in the anterior part (dynamic component of the weight-bearing force); and biconcave fractures (fishtail shape), which are most common in the lumbar spine and are when the vertebra collapses medially (static component of the force). Wedge fractures are the most common, followed by biconcave, compression, and then a combination of all three types of fractures. An upright gait compresses the vertebra to the density required by the body mass (in the numerator) according to the Nevitt coefficient (in the denominator). This explains more than 60% of vertebral fractures in which the patient has no memory of the incident (Fig. 1).

Recent studies suggest that spontaneously occurring fractures of the thoracolumbar spine that are initially asymptomatic account for more than 60%. When conventional radiology shows no traumatic deformity of the vertebra, MRI is used to detect occult fractures. In an elderly person, a small external force, for example, a fall from standing height or lifting a moderately heavy load, causes a fracture in the thoracolumbar spine. The Nevitt bone strength factor explains the occurrence of a VFC even in the absence of an accidental event [4].

## Classification of thoracolumbar spine fractures in osteoporosis

There are several classifications [5]. The AO spine thoracolumbar classification system is a synthesis of clinical and imaging investigations and explains the deformity of the bone at the time of fracture by the direction of the force applied to the vertebra and considers any clinical modifiers. It classifies fractures into A type (axial compression with intact posterior constraining elements), type B (failure of the posterior constraining elements) and type C (failure of anterior and posterior elements leading to displacement). In type A, the posterior elements are not affected; subgroups A 1 to 4 (wedge, split or pincer-type impaction fractures, posterior wall incomplete and subtype A4 complete burst fractures) consider the involvement of the posterior wall and both terminal plates of the vertebra. In type B, fractures without displacement of the spinal axis consider the distraction of the posterior elements of the tensile column (laminae, spinous processes, ligaments); and in fractures with displacement of the spinal axis, which are unstable, there is a translational injury of group C.

Fractures due to osteoporosis are generally not neuro-aggressive, as the vertebra collapses in on itself. The German Association for Traumatology and Orthopaedics DGOU divides osteoporotic fractures into five groups:

- Type 1: MRI-confirmed oedema in the vertebra, without visible fracture (occult fracture according to Genant).
- Type 2: fracture without posterior wall involvement.
- Type 3: fracture where the fracture exhibits a distinct involvement of the posterior wall.
- Type 4: loss of integrity of the vertebral frame or vertebral body collapse.
- Type 5: fractures with distraction or rotation [6].

## Diagnostic treatment

After a detailed medical history, information on high- or low-energy trauma, the mechanism of fracture (trying to identify the parallelogram of forces acting on the spine), comorbidities, and other relevant information, a clinical examination of the patient (frontal and sagittal spinal axis) follows. Important parameters are:

- Their ability to be independent,
- Their upright posture and gait,
- Where pain is felt and the direction of pain,
- Information on height loss,
- Possible transverse skin folds on the back,
- Neurological and circulatory distal abnormalities,
- Symptoms and signs of comorbidities (disturbance of consciousness, intoxication, effect of medication, haemodynamic stability, etc.),
- Information on possible osteoporosis already diagnosed, DXA scan, VFA (vertebral fracture assessment), staged imaging with dual-plane X-ray, in case of ambiguity CT, MR imaging, CT angiography.

## Therapy

In the past, the treatment of choice has been conservative treatment with analgesic therapy, early mobilisation, and functional treatment with physical therapy with exercises to strengthen the muscular corset. The decision on the treatment modality is made after a precise definition of the fracture with an assessment of the angulation of the spine after the injury, the degree of comminution of the vertebral body, the involvement of the neurological structures in the spinal canal, and concomitant diseases and defects of the spinal column due to other pre-existing diseases.

### Conservative therapy

Conservative treatment for osteoporotic thoracolumbar spinal fractures typically involves bed rest (adequate for a very short period), pain management, and physical therapy. The goal of conservative treatment is to relieve pain, prevent further vertebral collapse, and promote healing. However, conservative treatment is not always effective, and patients may require surgical intervention to achieve adequate pain relief and improve mobility.

Stable type A fractures without posterior ligament damage are, in most cases, treated conservatively with functional treatment and adequate analgesia. Treatment with orthoses is not a good option, although orthosis has an analgesic effect. With the use of orthoses, a greater loss of muscle mass and muscle strength is expected in the muscles along the spine and in the muscular corset. With conservative therapy, any additional vertebral subsidence should be monitored with standing X-rays at two-to-three-week intervals until fracture repair. We recommend offloading the vertebra with crutches during the healing period. In young and middle-aged people, kyphotic angulation of more than 20° is an indication for surgical treatment. In the elderly, we assess the general condition of the patient, the comorbidities, the expected survival, or the patient's expectations and requirements concerning quality of life. If concomitant injuries of the adjacent intervertebral discs are found, careful follow-up of the treatment is necessary, as the likelihood of neurological impairment is higher. (This increases the indication for surgical treatment. In young and middle-aged people, short segment posterior stabilisation is usually sufficient in the presence of good bone strength. In osteoporotic bone, longer stabilisation is required, at least of two adjacent upper and two adjacent lower vertebrae. In the elderly, anterior reconstruction is rarely indicated, in case of compromise of adjacent structures and organs).

In the case of unclear anamnestic data, low-energy trauma or even no history of trauma, and spontaneously occurring spinal pain, other pathological causes of vertebral body subsidence (metastases, plasmocytoma, infection, etc.) should be excluded. If the cause of the spinal pain and spinal deformity is unclear, the patient should also be referred to a physician who will clinically identify the underlying disease in the laboratory and with the Fracture Risk Assessment Tool (Frax) programme. When deciding on conservative treatment, we recommend a DGOU scoring system of 1 to 5. When monitoring vertebral subsidence in advanced osteoporosis, we use the Genant fracture classification (mild, moderate, severe) to monitor VFA by densitometry. Most osteoporotic fractures in the thoracolumbar segment are treated conservatively with appropriate pain management, activity modification, crutches, orthotics, and physical therapy. Conservatively treated patients with osteoporotic fractures of the thoracolumbar spine should be followed up clinically and radiologically even after fracture repair, as the process of subsidence due to osteoporosis is continuous. In the case of intractable acute pain, persistence of severe pain for more than six to twelve weeks or progression of local kyphosis, conservative treatment can be combined with posterior injection of bone cement into the vertebral body, vertebroplasty or balloon kyphoplasty, which are minimally invasive posterior methods.

## Balloon kyphoplasty and vertebroplasty

Balloon kyphoplasty (BK) and vertebroplasty (VP) are two minimally invasive augmentation techniques that have been critically scrutinised since 2009 following two articles that raised doubts in the scientific community (sham control studies). Consequently, the number of procedures performed has declined in the following few years, even leading to reduced survival of patients. Five-year follow-up of patients in 2009 was associated with an increased risk of mortality [7].

Subsequent studies have proven the effectiveness of both augmentation methods, and recommendations again classify them as effective methods for the treatment of acute fracture pain, chronic pain, and a proven increase in the quality of life of an elderly person with a fracture. In contrast to non-surgical treatment, BK is more effective in reducing pain, “back-related disability”, and improving quality of life [8].

BK is a minimally invasive surgical procedure for the treatment of pain and correction of the kyphotic angle in osteoporotic fractures of type A1 and A2. Two inflatable balloons, introduced transpedicular, are used to correct the vertebral deformity and to fill the fracture fissures or the cavity created by the inflated balloon in the vertebra with cement (“eggshell” technique). The available literature focuses on the “pain killing” effect, and the elderly person is generally able to stand upright on their own for a few hours after the procedure, without any significant pain. Indications for BK are:

- Unbearable pain from an acute fracture of the thoracic or lumbar spine.
- A tendency for continuous vertebral collapse and additional loss of height, visible on standing radiographs.
- Persistence of acute pain for weeks after the fracture; some authors take three weeks as the limit, whereas others use six to twelve.

In osteoporotic fractures, it is sometimes difficult to distinguish a fresh fracture from previous chronic lesions and acute fracture pain from other medical causes, so routine MRI of the spine is advised before BK.

Vertebroplasty (VP) is an augmentation method where cement is also injected transpedicular into the fractured vertebral body, but without inflatable balloons; however, the Cobb angle of kyphosis is not corrected as much as in BK. Some authors consider VP as a developmental precursor of BK.

Both methods have described complications, such as extravasation of cement through fracture fissures from the vertebral body into adjacent anatomical structures (spinal canal, vena cava, aorta), compression of neurological structures, and venous embolisms. The “eggshell” technique often avoids these complications in BK. Comparison of late results one year after VP and BK revealed that the fracture of the adjacent vertebra occurs according to the progression of osteoporosis and according to the biomechanics of kyphosis. By both methods, adjacent vertebral fracture represents a rare complication, with no statistically significant differences in incidence. The results of the studies show that adjacent vertebral fracture is likely even without an adjacent vertebral augmentation procedure. Both methods offer comparable pain reduction [9].

Balloon kyphoplasty is preferred for fractures with more than 50% loss of vertebral height, as it allows for greater vertebral height restoration and improved alignment. In contrast, vertebroplasty is preferred for fractures with less than 50% loss of vertebral height.

Recent studies have shown that both vertebroplasty and balloon kyphoplasty are safe and effective procedures for selected patients with osteoporotic thoracolumbar spinal fractures associated with significant improvements in pain relief, mobility, and quality of life compared to conservative treatment.

Minimally invasive techniques such as vertebroplasty and balloon kyphoplasty offer several advantages over traditional open surgical procedures. These procedures can be performed under local anaesthesia and conscious sedation, avoiding the risks associated with general anaesthesia. They also involve smaller incisions, reducing the risk of postoperative complications such as wound infection and bleeding. Moreover, these procedures can be performed as outpatient procedures, allowing patients to return home on the same day.

## Surgical treatment

However, it is important to note that vertebroplasty and balloon kyphoplasty are not suitable for all patients with osteoporotic thoracolumbar spinal fractures. Patients with significant spinal deformity or those with multiple vertebral fractures may require more extensive surgical intervention.

Additionally, these procedures carry the risk of complications such as cement extravasation, which can lead to nerve damage and other complications.

Posterior spinal instrumentation, on the other hand, is a surgical procedure that involves the placement of screws, rods, or other hardware along the back of the spine to stabilise and support the spine. By stabilising the spine, posterior spinal instrumentation can help to reduce pain, improve mobility, and prevent further damage to the spine.

**Fig. 2:**
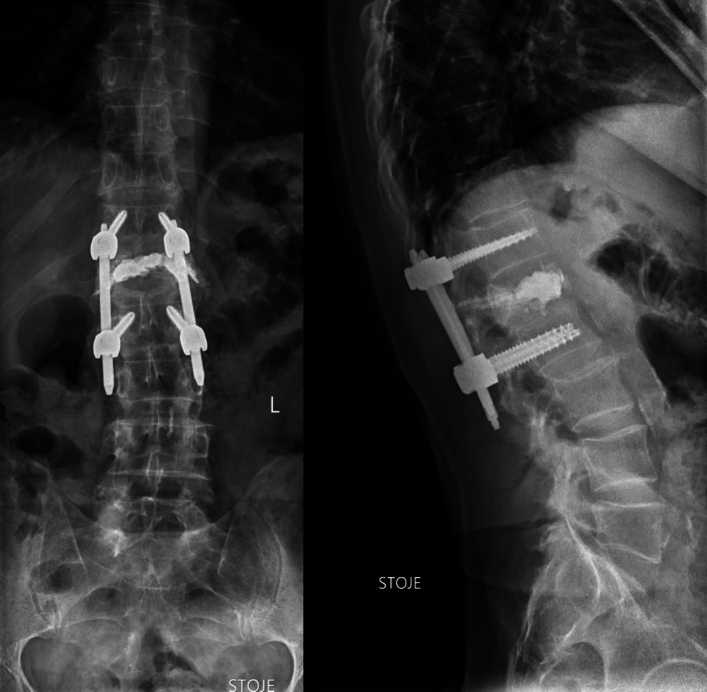
BK plus posterior instrumentation

Balloon Kyphoplasty (BK) and posterior spinal instrumentation are two procedures that are often performed together to treat certain spinal conditions (Fig. 2).

While each of these procedures can be performed individually, combining them can provide a more comprehensive solution for certain patients.

**Fig. 3:**
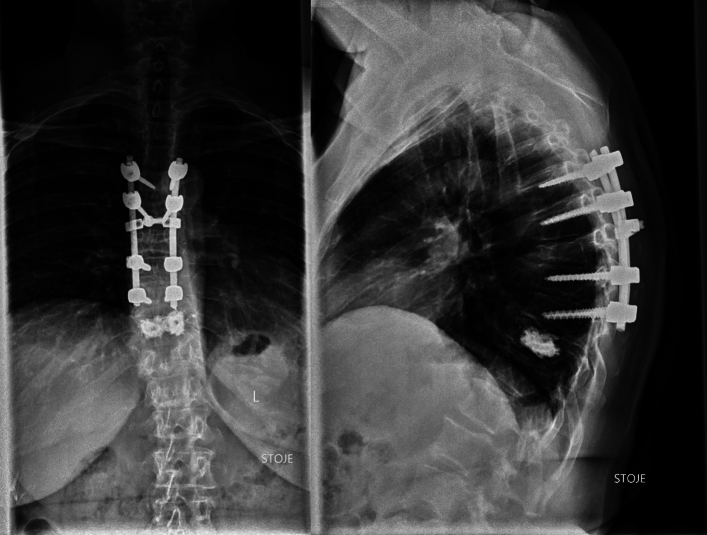
BK and instrumentation at the same time

While both balloon kyphoplasty and posterior spinal instrumentation can be effective treatments on their own, there are situations with combining the two procedures may be necessary. For example, if a patient has a vertebral compression fracture that is causing spinal deformity, a combination of balloon kyphoplasty and posterior spinal instrumentation may be the best solution. Balloon kyphoplasty can restore the height of the fractured vertebrae and reduce the deformity, while posterior spinal instrumentation can provide additional support to the spine to prevent further damage or deformity (Fig. 3).

**Fig. 4:**
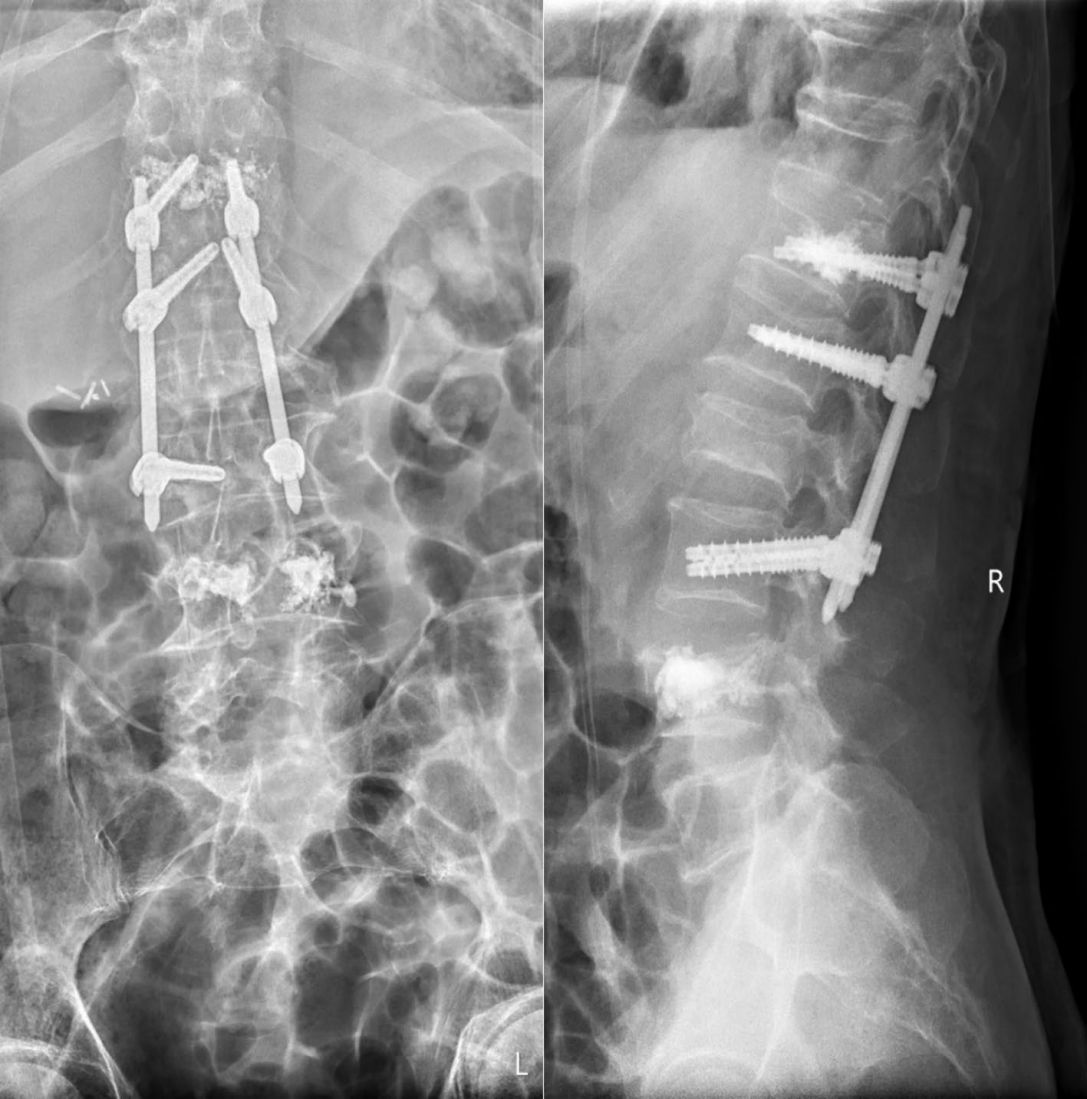
BK, followed by instrumentation due to exacerbation of osteoporosis

Another situation with combining balloon kyphoplasty and posterior spinal instrumentation may be appropriate when a patient has multiple vertebral compression fractures. In this case, balloon kyphoplasty can be used to treat each of the individual fractures, while posterior spinal instrumentation can be used to stabilise the entire spine and prevent further fractures from occurring. The quality of bone tissue may compromise the stability of the screws, that’s why the screws augmented with cement can additionally stabilize the instrumentation (Fig. 4).

**Fig. 5:**
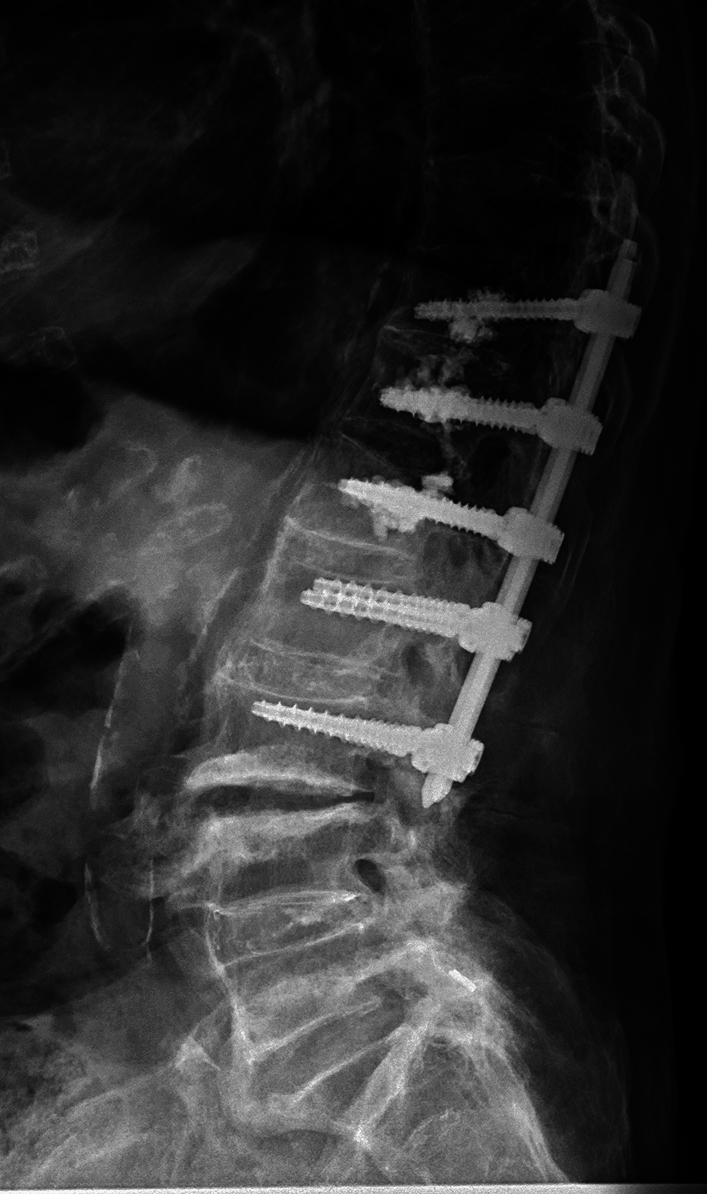
Augmented screws

**Fig. 6:**
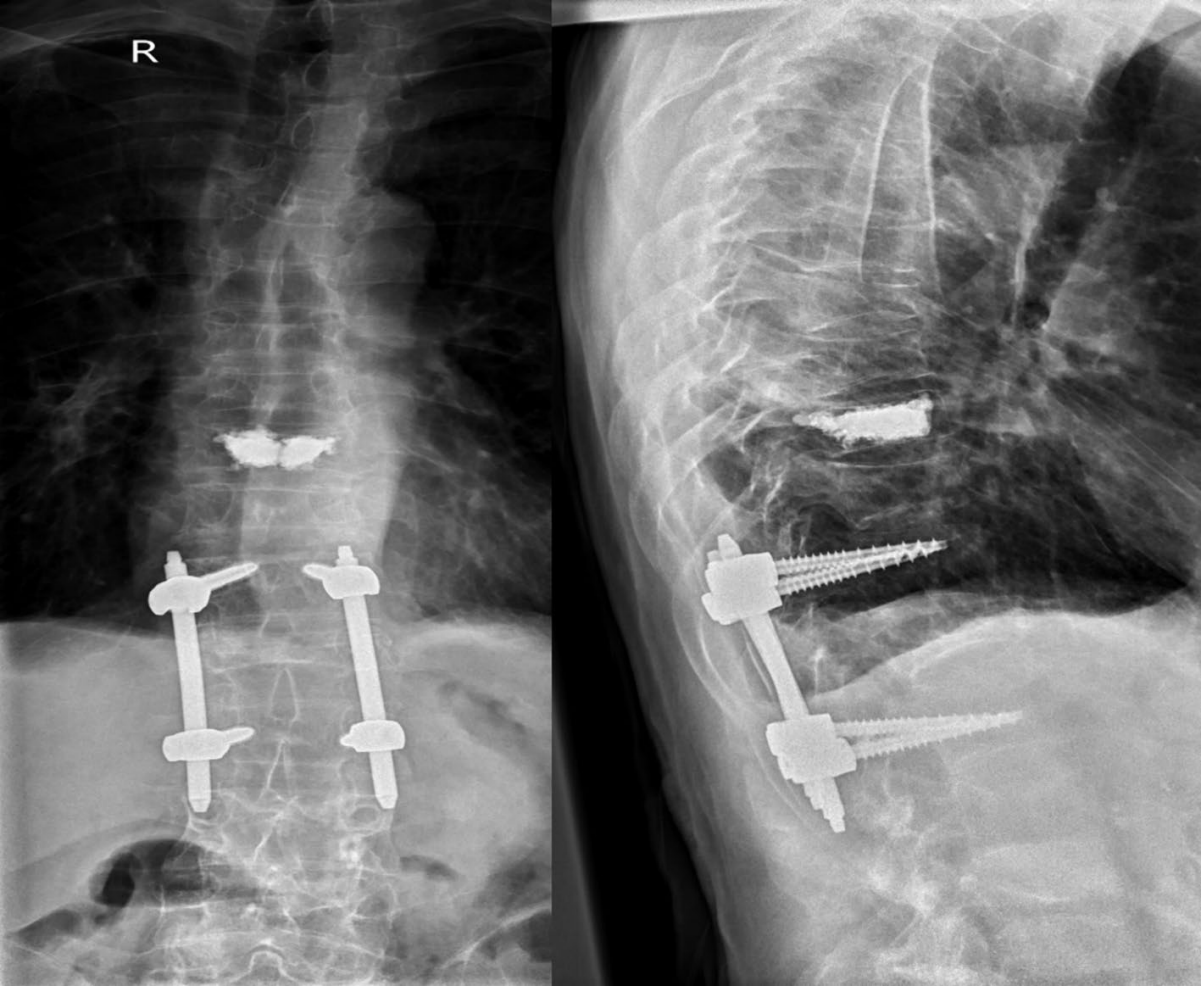
Combination of multiple fractures

It is important to note that not all patients with vertebral compression fractures or other spinal conditions will require both balloon kyphoplasty and posterior spinal instrumentation. Each patient's situation is unique, and the best treatment plan will depend on a variety of factors, including the location and severity of the condition, the patient's overall health, and their individual needs and preferences (Figs. 5, 6).

In conclusion, balloon kyphoplasty and posterior spinal instrumentation are two procedures that can be effective treatments for certain spinal conditions. While they can be performed individually, combining the two procedures may provide a more comprehensive solution for certain patients.

Photo material from the spinal team at the GH Celje, Slovenia.

## Data Availability

No datasets were generated or analysed during the current study.
